# Libanoridin Isolated from *Corydalis heterocarpa* Inhibits Adipogenic Differentiation of Bone Marrow-Derived Mesenchymal Stromal Cells

**DOI:** 10.3390/ijms24010254

**Published:** 2022-12-23

**Authors:** Fatih Karadeniz, Jung Hwan Oh, Mi Soon Jang, Youngwan Seo, Chang-Suk Kong

**Affiliations:** 1 Marine Biotechnology Center for Pharmaceuticals and Foods, College of Medical and Life Sciences, Silla University, Busan 46958, Republic of Korea; 2 Nutritional Education, Graduate School of Education, Silla University, Busan 46958, Republic of Korea; 3 Food Safety and Processing Research Division, National Institute of Fisheries Science, Busan 46083, Republic of Korea; 4 Division of Convergence on Marine Science, College of Ocean Science and Technology, Korea Maritime and Ocean University, Busan 49112, Republic of Korea; 5 Department of Food and Nutrition, College of Medical and Life Sciences, Silla University, Busan 46958, Republic of Korea

**Keywords:** adipogenesis, bone marrow mesenchymal stromal cells, *Corydalis heterocarpa*, libanoridin, PPARγ

## Abstract

Bone marrow adiposity is a complication in osteoporotic patients. It is a result of the imbalance between adipogenic and osteogenic differentiation of bone marrow cells. Phytochemicals can alleviate osteoporotic complications by hindering bone loss and decreasing bone marrow adiposity. *Corydalis heterocarpa* is a biennial halophyte with reported bioactivities, and it is a source of different coumarin derivatives. Libanoridin is a coumarin isolated from *C. heterocarpa,* and the effect of libanoridin on adipogenic differentiation of human bone marrow-derived mesenchymal stromal cells (hBM-MSCs) was evaluated in the present study. Cells were induced to undergo adipogenesis, and their intracellular lipid accumulation and expression of adipogenic markers were observed under libanoridin treatment. Results showed that 10 μM libanoridin-treated adipocytes accumulated 44.94% less lipid compared to untreated adipocytes. In addition, mRNA levels of PPARγ, C/EBPα, and SREBP1c were dose-dependently suppressed with libanoridin treatment, whereas only protein levels of PPARγ were decreased in the presence of libanoridin. Fluorescence staining of adipocytes also revealed that cells treated with 10 μM libanoridin expressed less PPARγ compared to untreated adipocytes. Protein levels of perilipin and leptin, markers of mature adipocytes, were also suppressed in adipocytes treated with 10 μM libanoridin. Analysis of MAPK phosphorylation levels showed that treatment with libanoridin inhibited the activation of p38 and JNK MAPKs observed by decreased levels of phosphorylated p38 and JNK protein. It was suggested that libanoridin inhibited adipogenic differentiation of hBM-MSCs via suppressing MAPK-mediated PPARγ signaling. Future studies revealing the anti-adipogenic effects of libanoridin in vivo and elucidating its action mechanism will pave the way for libanoridin to be utilized as a nutraceutical with anti-osteoporotic properties.

## 1. Introduction

Bone marrow adipose tissue is one of the fat deposits in the human body and is critically involved in bone homeostasis and hematopoiesis [1]. Metabolic disorders such as obesity and osteoporosis are intertwined through complex biological processes, but one of the common complications between osteoporosis and obesity is the excessive adiposity in bone marrow [2,3]. Increased adiposity does affect not only fat accumulation but also the secretion of adipogenic hormones [4]. Patients suffering from obesity and osteoporosis were found to host more extensive bone marrow adipose tissue than healthy individuals [3,5]. Osteoporosis is one of the most prevalent bone disorders affecting men and women globally [6]. The high prevalence of osteoporosis and its detrimental effect on life quality urge researchers to find ways to prevent or treat osteoporotic complications.

Osteoporotic complications arise from a deteriorating balance between adiposity and bone formation. Mesenchymal stromal cells in the bone marrow are cells with multipotent differentiation capacity into adipocyte and osteoblast progenitors [7]. Under osteoporotic conditions, differentiation tendencies of bone marrow mesenchymal stromal cells favor adipogenesis instead of osteoblastogenesis [8]. This imbalance leads to increased bone marrow adiposity, one of the reasons behind bone loss and fragility, in addition to dysregulation of adipose tissue-related hormonal balance [9,10]. Like other adipose tissues, such as white and brown, bone marrow adipogenesis follows a similar signaling cascade for initiating differentiation into adipocyte progenitor cells and subsequent maturation into adipocytes [11]. Differentiation of human bone marrow mesenchymal stromal cells (hBM-MSCs) into adipocytes is a strictly regulated progress mainly controlled by the expression and transcriptional activity of peroxisome proliferator-activated receptor gamma (PPARγ) along with CCAAT/enhancer-binding proteins (C/EBP) and sterol regulatory element-binding protein 1c (SREBP1c) [12,13]. Upregulation of these transcription factors stimulates adipogenic differentiation.

There are many reported ways to relieve or prevent osteoporotic complications, such as increasing bone formation [14], hindering bone loss [15], and limiting bone resorption [16]. In this context, suppressing the adipogenic differentiation of hBM-MSCs was shown to tilt the differentiation balance toward osteoblastogenesis and prevent bone marrow adiposity while increasing bone formation [17,18]. Phytochemicals have been studied immensely in this regard due to their biocompatibility and diversity and assumed fewer side effects to treat osteoporosis. To date, phytochemicals have been reported to inhibit adipogenesis in different types of cells, during different stages of adipogenesis, and via different action mechanisms [19,20,21]. As a part of an ongoing effort to discover anti-osteoporotic substances, *Corydalis heterocarpa* was investigated to isolate bioactive compounds. *C. heterocarpa* is a biennial halophyte found throughout Asia. Reports have shown that *C. heterocarpa* might contain anti-inflammatory [22], antitumor [23], antioxidant [24], and anti-adipogenic [25] agents. Previous studies reported bioactive coumarins such as heterocarpin, hyunganol-II, columbianetin, and libanoridin isolated from *C. heterocarpa* as active ingredients [22,25,26]. The present study investigated the anti-adipogenic potential of libanoridin isolated from *C. heterocarpa* in human bone marrow-derived mesenchymal stromal cells (hBM-MSCs). To the best of our knowledge, this is the first report that shows the adipogenesis inhibitory effect of libanoridin.

## 2. Results

### 2.1. Inhibition of Lipid Accumulation

**Figure 1:**
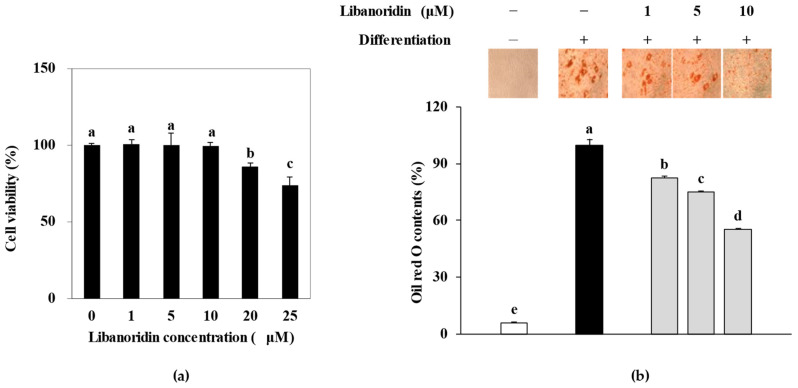
Effect of libanoridin on viability and lipid accumulation in hBM-MSCs. (**a**) Effect of libanoridin on the viability of non-differentiated hBM-MSCs. Cells were treated with libanoridin for 72 h. Cell viability is given as a relative percentage of the untreated (0 μM) group. (**b**) Effect of libanoridin on the amount of accumulated intracellular lipid droplets. Droplets were stained by Oil Red O at day 12 of differentiation, and the Oil Red O stain retained by the cells was given as a relative percentage of the differentiated untreated group. Values are means ± SD (*n* = 3). ^a–e^ Bars with identical letters indicate no statistical significance (*p* < 0.05), while different letters indicate otherwise.

Different concentrations of libanoridin (0, 5, 10, 20, and 25 μM) were added to the growth medium of hBM-MSCs. The cells were incubated for 72 h to evaluate any toxic presence of libanoridin prior to anti-adipogenesis activity screening. The first significant decrease in viable cell levels was recorded for doses above 10 μM (Figure 1a). Therefore, subsequent experiments were carried out using libanoridin concentrations up to 10 μM. In order to evaluate its effect on hBM-MSC adipogenesis, libanoridin was present with the initial adipogenic differentiation medium fed to cells for 3 days, followed by a fresh medium change. According to cytotoxicity analysis, any decrease in adipogenic differentiation was not due to the cytotoxicity of libanoridin.

The adipogenic differentiation of hBM-MSCs was confirmed by Oil Red O staining of intracellular lipid deposits. The main characteristic of mature adipocytes is intracellular lipid storages shaped as droplets to store triglycerides. Staining results showed that adipo-induced hBM-MSCs accumulated a notable amount of lipid droplets compared to non-differentiated cells (Figure 1b). Treatment with libanoridin dose-dependently decreased the lipid droplet amount. Measurement of the stain retained intracellularly further confirmed this. At the concentration of 10 μM, libanoridin treatment decreased the intracellular lipid amount by 44.94%. This ratio was 24.86% and 17.49% for 5 and 1 μM treatment, respectively. These results suggested that libanoridin treatment hindered the adipogenic differentiation of adipo-induced hBM-MSCs. Studies showed that lipid accumulation inhibition mostly translated into suppressed adipogenesis or stimulated lipid hydrolysis, suggesting anti-adipogenic activity against bone marrow adiposity.

### 2.2. Supression of PPARγ Expression

In order to evaluate whether the inhibitory effect of libanoridin on lipid accumulation of adipo-induced hBM-MSCs was a result of suppressed adipogenesis, the expression of adipogenic transcription factors was evaluated. For this analysis, genistein was used as a positive control. Genistein is a phytoestrogen and chemically very similar to estrogens, which is why it was found to exhibit beneficial effects in osteoporosis [27]. Genistein was shown to possess bone-protective properties by enhancing the osteoblastogenic differentiation of hBM-MSCs while repressing adipogenesis [27,28]. The transcription factor PPARγ is at the center of adipogenic differentiation of all types of adipose tissue, varying from white and brown to bone marrow and beige [12,13]. SREBP1c stimulates its transcriptional activity along with C/EBPα, which, together with PPARγ, regulates the growth arrest and differentiation of committed pre-adipocytes [13].

**Figure 2:**
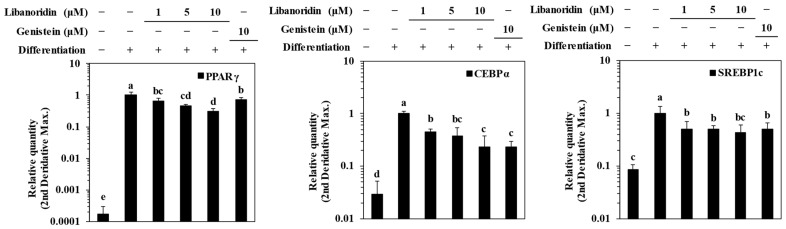
Effect of libanoridin on the mRNA expression of adipogenic transcription factors: PPARγ, C/EBPα, and SREBP1c. Analysis of the expression levels was carried out via RT-qPCR on adipo-induced hBM-MSCs at day 7 of differentiation. Values are means ± SD (*n* = 3). ^a–e^ Bars with identical letters indicate no statistical significance (*p* < 0.05), while different letters indicate otherwise.

**Figure 3:**
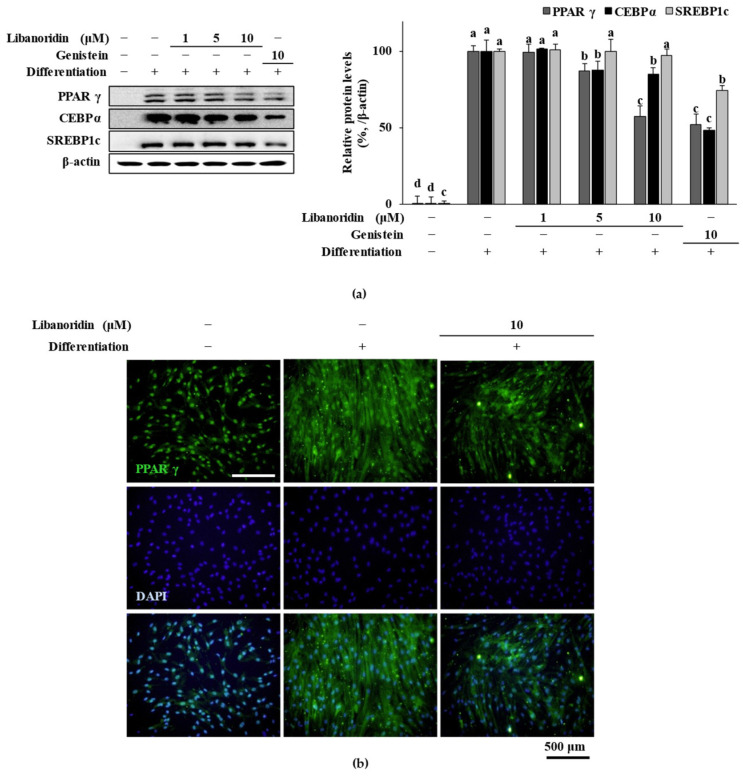
Effect of libanoridin on protein levels of adipogenic transcription factors. (**a**) Protein expression levels of PPARγ, C/EBPα, and SREBP1c in adipo-induced hBM-MSCs were analyzed by Western blotting at day 7 of differentiation. β-actin was used as an internal loading control. Values are means ± SD (*n* = 3). ^a–d^ Bars with identical letters indicate no statistical significance (*p* < 0.05), while different letters indicate otherwise. (**b**) The intracellular PPARγ protein levels were investigated by fluorescence staining (green). DAPI staining was employed (blue) to highlight the viable cell nucleus.

Results showed that mRNA expression levels of PPARγ, C/EBPα, and SREBP1c were all significantly increased in adipo-induced hBM-MSCs (Figure 2). Treatment with libanoridin suppressed the mRNA expression levels of all three adipogenic marker mRNAs. On the other hand, genistein (10 μM) also showed a similar effect to that of libanoridin at 10 μM for C/EBPα and SREBP1c. At the same time, its suppression of PPARγ mRNA levels was lower than libanoridin. Western blot analysis further confirmed the inhibitory effect of libanoridin on PPARγ expression. Protein levels of PPARγ, C/EBPα, and SREBP1c were all notably elevated in adipo-induced hBM-MSCs (Figure 3a). Libanoridin treatment was able to decrease the PPARγ levels significantly at 10 μM concentration. However, its effect on SREBP1c was not significant where genistein significantly decreased SREBP1c levels, and although it decreased C/EBPα levels, the effect was not comparable to that of genistein (10 μM).

Nevertheless, fluorescence staining of intracellular PPARγ levels showed that libanoridin (10 μM) significantly suppressed the levels of PPARγ in adipo-induced hBM-MSCs suggesting that it repressed the adipogenesis via PPARγ-related pathway (Figure 3b). Results showed that the effect of libanoridin on mRNA levels was translated into protein levels of PPARγ and C/EBPα at 5 and 10 μg/mL treatment but did not affect SREBP1c levels indicating that the anti-adipogenic effect of libanoridin was exhibited through suppressing the adipogenic marker proteins of mature adipocytes. In this case, unaffected protein levels of SREBP1c would initiate adipogenesis, but the cells would not express adipogenic characteristics, which progress during maturation.

### 2.3. Suppression of Perilipin and Leptin Levels

PPARγ is a critical transcription factor in adipogenesis but also takes roles in other intracellular reactions and can be found in tissues other than adipose [29]. Following the effect of libanoridin on PPARγ levels, other adipogenic markers were tested to confirm further that the effect observed in suppressing PPARγ levels was related to adipogenesis but not basal PPARγ expression. In addition, the inability of libanoridin to reduce the protein expression of SREBP1c indicated that it might alter adipogenesis through other pathways. In this context, we have investigated the expression of perilipin and leptin, both present in mature adipocytes in hBM-MSCs. Perilipin, also known as lipid droplet-associated protein, is the protein in adipocytes encapsulating the intracellular lipid droplets. It is located on the surface of lipid droplets and is crucial for lipolysis regulation [30]. Furthermore, leptin is an adipokine: a hormone produced and secreted by the adipose tissue regulating body weight [31].

**Figure 4:**
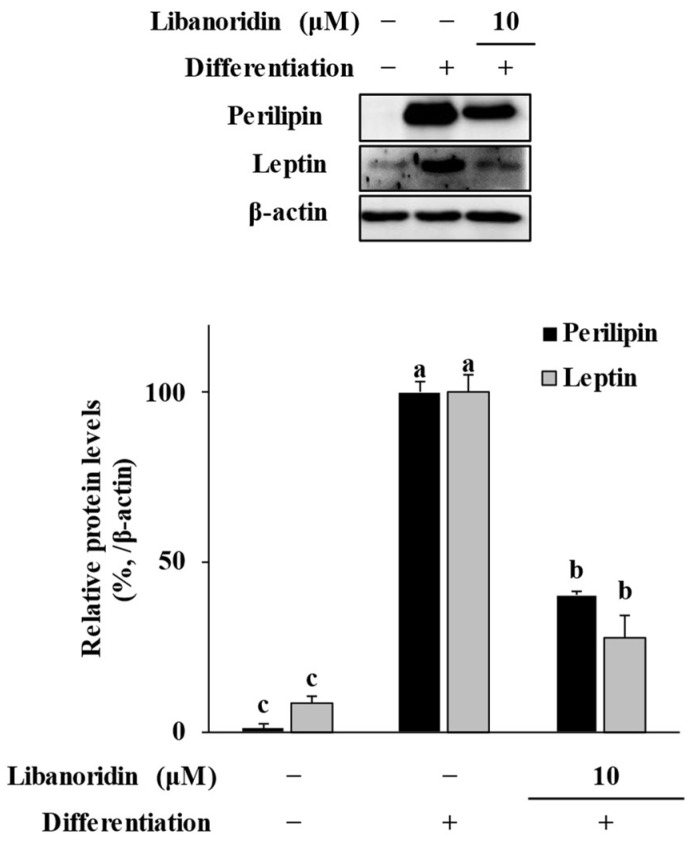
Effect of libanoridin on protein levels of adipogenic markers: perilipin and leptin. Protein expression levels in adipo-induced hBM-MSCs were analyzed by Western blotting at day 12 of differentiation. β-actin was used as an internal loading control. Values are means ± SD (*n* = 3). ^a–c^ Bars with identical letters indicate no statistical significance (*p* < 0.05), while different letters indicate otherwise.

The protein levels of perilipin and leptin were investigated in hBM-MSC adipocytes in the presence or absence of libanoridin (10 μM) by Western blot analysis. Adipo-induced cells expressed significant levels of perilipin and leptin as a marker of adipogenic maturation (Figure 4). Treatment with libanoridin significantly reduced the levels of perilipin and leptin, suggesting that the effect of libanoridin on PPARγ levels indicated an anti-adipogenic activity. However, libanoridin did not alter protein expression of adipogenic transcription factors except for PPARγ, and it exerted an anti-adipogenic activity decreasing adipogenic characteristics of adipo-induced hBM-MSCs.

### 2.4. Repression of MAPK Activation

**Figure 5:**
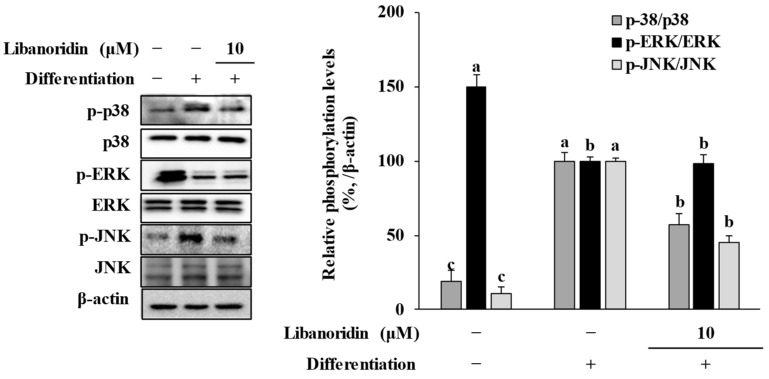
Effect of libanoridin on the activation of MAPKs. The total and phosphorylated (p-) levels of p38, ERK, and JNK MAPKs were investigated in adipo-induced hBM-MSCs at day 12 of differentiation by Western blot. β-actin was used as an internal loading control. Values are means ± SD (*n* = 3). ^a–c^ Bars with identical letters indicate no statistical significance (*p* < 0.05), while different letters indicate otherwise.

The critical involvement of MAPK activation during all stages of adipogenesis is known, and studies showed that several anti-adipogenic substances exert their effect via MAPK signaling-mediated suppression of PPARγ activation [25,30,31,32,33]. Stimulation of adipogenesis was shown to be related to transcriptional activities of AP-1 and CREB transcription factors, both of which are translocated into the nucleus after phosphorylation by MAPKs [34,35]. In this context, phosphorylation of three MAPKs: p38, ERK, and JNK, were evaluated by Western blot in adipo-induced hBM-MSCs. Results showed that hBM-MSC adipocytes showed increased phosphorylation of p38 and JNK compared to non-differentiated cells, while phosphorylation of ERK was suppressed (Figure 5). The presence of 10 μM libanoridin suppressed the adipogenesis-related p38 and JNK phosphorylation but did not alter ERK activation. Studies reported controversial results for the role of p38 in adipogenesis. While some reports suggested that suppressing p38 MAPK stimulates adipogenesis [36,37], opposite results were also presented where activation of p38 MAPK is required for adipogenesis [38,39,40,41]. Current results showed that hBM-MSCs exhibited increased p38 activation, which was suppressed by libanoridin. The effect of libanoridin on PPARγ activation might be linked with suppressed p38 activation in the earlier stages of adipogenesis, considering the effect on PPARγ but not SREBP1c. Similar contradictory reports could also be found for the role of JNK. Although current results showed that adipo-induced hBM-MSCs required activation of JNK, which is consistent with other studies reporting anti-adipogenic agents that suppress JNK activation [42,43,44], some studies reported that suppressed JNK activation promotes adipogenic differentiation [45,46].

Nevertheless, present study results showed that libanoridin presence relieved the activation levels of p38 and JNK, which were shown to be stimulated upon inducing adipogenesis, suggesting that this might be associated with the inhibitory effect of libanoridin on suppression of PPARγ expression. Similar results were obtained in a recent report on the anti-adipogenic potential of hyunganol-II. Hyunganol-II is a bioactive coumarin similar to libanoridin and isolated from the same source (*C. heterocarpa*). In Oh et al. [25] study, hyunganol-II suppressed the adipogenic differentiation of hBM-MSCs via suppression of p38 and JNK activation. Parallel to current results, hyunganol-II did not alter protein levels of SREBP1c. However, hyunganol-II stimulated ERK activation, which was not affected by libanoridin treatment. Their coumarin structural background might be the reason for their anti-adipogenic activity, considering the similar action mechanism of libanoridin and hyunganol-II. Further, *C. heterocarpa* might be a source of anti-adipogenic agents. In either case, future investigations are necessary to characterize and clarify the roles of MAPKs in different stages of hBM-MSC adipogenesis regarding their suppression or activation via coumarin derivatives.

In conclusion, the present study showed that libanoridin inhibited adipogenic differentiation in hBM-MSCs via inhibition of p38 and JNK MAPK phosphorylation, which suggestively suppressed the PPARγ expression resulting in repressed adipogenesis. The hBM-MSCs were induced to differentiate into adipocytes in the presence or absence of libanoridin. Cells with libanoridin treatment showed decreased levels of PPARγ in both mRNA and protein levels. In addition, protein levels of other adipogenic markers, perilipin, and leptin, were significantly decreased upon libanoridin treatment compared to untreated adipo-induced cells. While adipo-induced cells expressed elevated levels of MAPK activation, libanoridin presence decreased the activation of p38 and JNK MAPK. Inhibiting PPARγ expression via MAPK suppression was suggested to be a potential mechanism by which libanoridin suppressed adipocyte formation in hBM-MSCs. Overall, these results indicate that libanoridin should be further evaluated as a potential anti-adipogenic agent to decrease bone marrow adiposity and relieve osteoporotic complications.

## 3. Materials and Methods

### 3.1. Materials

Libanoridin was isolated from the whole plants of *C. heterocarpa,* which was handpicked in Muanda, Jeollanamdo, Republic of Korea. Whole parts of plants were dried under shade at room temperature and extracted by keeping each in MeOH and CH_2_Cl_2_ for two days, each solvent with occasional stirring. Crude extracts were collected following in vacuo evaporation of the solvents. Libanoridin was isolated from 85% aq. MeOH fraction of the combined crude extracts as a white amorphous solid. The isolation scheme and other details were reported earlier [22]. The recorded ^1^H and ^13^C NMRs, given below, were compared with published literature to identify the isolated compound as libanoridin.

Libanoridin: 127–129 °C; [α]^D^_25_ + 252 (c 1.0, CHCl_3_); HREI-MS *m*/*z* 288.0998 (calcd. for C_16_H_16_O_5_, 288.0998); ^1^H NMR (300 MHz, CDCl_3_) *δ*: 7.60 (^1^H, d, *J* = 9.6 Hz, H-4), 7.24 (^1^H, d, *J* = 8.3 Hz, H-5), 6.72 (^1^H, d, *J* = 8.3 Hz, H-6), 6.18 (^1^H, d, *J* = 9.6 Hz, H-3), 5.12 (^1^H, dd, *J* = 9.6, 7.8 Hz, H-2′), 3.35 (^1^H, dd, *J* = 16.5, 9.6 Hz, H-1′a), 3.26 (^1^H, dd, *J* = 16.5, 7.8 Hz, H-1′b), 1.97 (3H, s, H-7′), 1.55 (3H, s, H-4′/-5′), 1.50 (3H, s, H-4′/-5′); ^13^C NMR (75 MHz, CDCl_3_) *δ*: 170.0 (C-6′), 163.6 (C-7), 160.8 (C-2), 151.0 (C-9), 143.8 (C-4), 128.7 (C-5), 113.3 (C-10), 112.9 (C-8), 112.1 (C-3), 106.6 (C-6), 88.6 (C-2′), 82.0 (C-3′), 27.6 (C-1′), 22.3 (C-7′), 22.0 (C-4′/-5′), 21.0 (C-4′/-5′).

The multipotent mesenchymal stromal cell line derived from human bone marrow (hBM-MSC), its culture medium (Mesenchymal Stem Cell Growth Medium, C-28009), and adipogenic differentiation medium (Mesenchymal Stem Cell Adipogenic Differentiation Medium 2, C-28016) were all from PromoCell (Heildelberg, Germany). All other essential reagents and materials used in cell culture and maintenance were purchased from Gibco BRL (New York, NY, USA) unless separately noted otherwise. 3-[4,5-dimethylthiazol-2-yl]-2,5-diphenyltetrazolium bromide (MTT), dimethyl sulfoxide (DMSO), and isopropanol were from Sigma-Aldrich (St. Louis, MO, USA). The AccuPrep Universal RNA extraction kit was purchased from Bioneer (Daejeon, Republic of Korea). The Cell Script All-in-One cDNA synthesis premix was obtained from CellSafe (Yongin, Republic of Korea). The NE-PER nuclear protein extraction kit, BCA protein assay kit for total protein quantification, and primary antibodies used in Western blot analysis against normal and phosphorylated (p-) JNK were from Thermo Fisher Scientific (Waltham, MA, USA). ECL kit for the Western blot band visualization was bought from Amersham Biosciences (Amersham, U.K.). Antibodies used in Western blot analysis to detect protein levels of PPARγ (#2443), CCAAT/enhancer-binding protein (C/EBP) α (#2295), p38 (#8690), p-p38 (#4511), ERK (#4695), and p-ERK (#4370) were purchased from Cell Signal Technology (Danvers, MA, USA). The primary antibody against SREBP1c (ab3259) was from Abcam (Cambridge, U.K.). Secondary antibodies for the hybridization of primary antibodies were purchased from Santa Cruz Biotechnology (Santa Cruz, CA, USA). For the immunocytochemical fluorescence staining, Alexa Fluor 488-conjugated secondary antibody (A-11008) was from Invitrogen (Waltham, MA, USA) while PPARγ (ab9256) primary antibody was from Abcam. The remaining necessary materials for fluorescence staining were purchased as a bundle (#12727) from Cell Signal Technology.

### 3.2. Maintenance and Differentiation of hBM-MSCs

The hBM-MSCs were cultured in transparent flat-bottom 6-well plates unless otherwise noted. Prior to inducing adipogenesis, cells were grown in a culture medium until they reached 100% confluency. Between all processes, cells were kept in 37 °C incubators, which were set to have humidified environment with 5% CO_2_ content. Adipogenesis was induced in hBM-MSCs that were grown to confluence, initially by swapping the culture medium with the differentiation medium. The differentiation medium was changed with a fresh one every third day until most of the cells exhibited mature adipocyte characteristics by accumulating lipid droplets. In order to have reliable and unified results, this period was set to be 12 days from the initial introduction of the differentiation medium. Libanoridin was added with the first differentiation medium in different final concentrations and was not present in subsequent medium changes. One group was induced to differentiate without libanoridin treatment, while one other group was kept in a culture medium without libanoridin treatment for comparison.

### 3.3. Assessment of Cytotoxicity

Prior to adipogenesis assays, toxic doses of libanoridin on non-differentiated hBM-MSCs were analyzed by MTT assay following a common procedure. Briefly, the hBM-MSCs were cultured until they reached approximately 80% confluency. Cells were then added with different concentrations of libanoridin (0~25 μM) in the growth medium. Treatment lasted for 72 h. After 72 h, the culture medium was swapped with fresh growing medium, which contained 0.2 mg/mL MTT. Cells were incubated for 4 h, and the wells were aspirated and supplied with 100% DMSO to stop the reaction and solubilize the formazan crystals. The optical density of the wells was then recorded at 540 nm with a MultiSkan GO microplate reader (Thermo Fisher Scientific). Viable cell levels in libanoridin-treated wells were calculated as a relative percentage of the untreated group.

### 3.4. Oil Red O Staining of the Intracellular Lipid Deposits

Lipid accumulation in the differentiated hBM-MSC adipocytes was observed and evaluated with Oil Red O staining. At the end of the differentiation period (day 12), cell culture wells were washed with PBS, and the adipocytes were fixed on wells by adding 1 mL formalin (neutral buffered, 10%) and incubating for 1 h. After fixation, formalin was aspirated, wells were air-dried, and 0.5% Oil Red O staining solution (wt/v, in 3 parts isopropanol and 2 parts triple-distilled water) was added to each well for staining. Staining lasted for 1 h at room temperature, after which wells were subsequentially aspirated, washed with PBS twice, and photographed. Lipid accumulation was also quantified by measuring the retained Oil Red O stain by intracellular lipid deposits. The stain was eluted from cells in each well by adding 1 mL of 10% isopropanol. Plates were then let rest at room temperature for the stain to be removed fully. The optical density of each well was then measured at 500 nm with a MultiSkan GO microplate reader. Obtained absorbance values were used to evaluate the lipid accumulation by plotting it as a relative percentage of the differentiated untreated hBM-MSC adipocytes.

### 3.5. Reverse Transcription Semi-Quantitative Polymerase Chain Reaction (RT-qPCR)

The expression levels of adipogenic transcription factors in differentiated hBM-MSCs were evaluated by RT-qPCR. On day 12 of differentiation total RNA content of each group was isolated with the AccuPrep Universal RNA extraction kit. Total RNA was treated with RNase-free DNase I to eliminate any DNA contamination and subsequently converted to cDNA using CellScript All-in-One cDNA master mix. Amplification of the adipogenic targets was carried out using common RT-qPCR procedures with specific primers, both of which were reported in detail earlier [25].

### 3.6. Immunoblotting

The protein expression levels of adipogenic transcription factors and MAPKs in differentiated hBM-MSC adipocytes were evaluated by Western blotting. On day 12 of differentiation, total protein content was isolated from cells. Briefly, cells were washed and then lysed with adding 1 mL RIPA buffer (supplemented with protease and phosphatase inhibitors) and pipetting. Obtained cell lysates were then separated by centrifugation (12,000 rpm, 10 min). Supernatants were used in Western blotting after their protein content was measured by a BCA protein assay kit. The same amount of protein from each group was then separated by SDS-PAGE (10%) and transferred onto membranes that were blocked for 4 h at room temperature with skim milk (5%). Membranes were then washed with TBS-T buffer, and the proteins were labeled with primary antibodies against target proteins overnight at 4 °C. Labeled proteins were then hybridized with horseradish peroxidase-conjugated secondary antibodies for 1 h at room temperature, and the protein bands were visualized with a commercial ECL kit and a chemiluminescence imager (CAS-400M, Davinch-K, Seoul, Republic of Korea).

### 3.7. Immunofluorescence Staining

Intracellular levels of the PPARγ, a key adipogenesis-inducing transcription factor, were also observed through fluorescence staining of the intracellular proteins. For this staining, hBM-MSCs were seeded, cultured and differentiated as described earlier but in 6-well plates that had glass coverslips placed at the bottom. At day 7 of differentiation, hBM-MSC adipocytes were fixed on wells with the addition of formalin (neutral buffered, 10%) for 1 h, and the formalin was aspirated. Cells were then loaded with Alexa Fluor 488 Green attached to the anti-PPARγ antibody. In addition, another set of cells was stained with ProLong Gold Antifade Reagent with 4′,6-diamidino-2-phenylindole (DAPI) to stain viable cell nuclei, necessary for normalization of the imaging. Images of the stained cells were taken after treatment with Immunofluorescence Application Solutions Kit (#12727; Cell Signaling Technology).

### 3.8. Statistical Analysis

All data are given as ± SD (*n* = 3) unless otherwise noted. The statistical significance of the results compared with the control group (differentiated untreated hBM-MSC adipocytes) was decided according to the results of a one-way analysis of variance followed by Duncan’s multiple range post hoc test. The least meaningful significance was determined at *p* < 0.05.

## Data Availability

The data presented in this study are available on request from the corresponding author.
